# Computer Vision and Machine Learning Analysis of Commercial Rice Grains: A Potential Digital Approach for Consumer Perception Studies

**DOI:** 10.3390/s21196354

**Published:** 2021-09-23

**Authors:** Aimi Aznan, Claudia Gonzalez Viejo, Alexis Pang, Sigfredo Fuentes

**Affiliations:** 1 Digital Agriculture, Food and Wine Group, School of Agriculture and Food, Faculty of Veterinary and Agricultural Sciences, University of Melbourne, Parkville, VIC 3010, Australia; aaznan@student.unimelb.edu.au (A.A.); cgonzalez2@unimelb.edu.au (C.G.V.); alexis.pang@unimelb.edu.au (A.P.); 2 Faculty of Chemical Engineering Technology, University Malaysia Perlis, Arau 02600, Perlis, Malaysia

**Keywords:** artificial neural networks, morpho-colorimetry, smartphone, photogrammetry, object of interest

## Abstract

Rice quality assessment is essential for meeting high-quality standards and consumer demands. However, challenges remain in developing cost-effective and rapid techniques to assess commercial rice grain quality traits. This paper presents the application of computer vision (CV) and machine learning (ML) to classify commercial rice samples based on dimensionless morphometric parameters and color parameters extracted using CV algorithms from digital images obtained from a smartphone camera. The artificial neural network (ANN) model was developed using nine morpho-colorimetric parameters to classify rice samples into 15 commercial rice types. Furthermore, the ANN models were deployed and evaluated on a different imaging system to simulate their practical applications under different conditions. Results showed that the best classification accuracy was obtained using the Bayesian Regularization (BR) algorithm of the ANN with ten hidden neurons at 91.6% (MSE = <0.01) and 88.5% (MSE = 0.01) for the training and testing stages, respectively, with an overall accuracy of 90.7% (Model 2). Deployment also showed high accuracy (93.9%) in the classification of the rice samples. The adoption by the industry of rapid, reliable, and accurate methods, such as those presented here, may allow the incorporation of different morpho-colorimetric traits in rice with consumer perception studies.

## 1. Introduction

Commercial rice (*Oryza sativa*) is available in various grades to meet consumer needs according to price and consumer preferences. The diverse rice germplasm consumed worldwide has high variability in its quality traits and has been linked with the physicochemical properties of the rice grains [1,2,3,4,5]. These traits are related to consumer acceptance of size and shape, color, odor/aroma, purity, homogeneity, and texture [6]. Raw rice quality is commonly associated with consumer perception, mainly before purchasing the product. It is evaluated visually based on the appearance of the rice grains, which is considered an important factor affecting buying decisions [7,8]. For example, the length, uniformity of size and shape, color, chalkiness, and percentage of broken rice were among the traits used to evaluate consumer perception of rice quality [6,7,8,9]. Meanwhile, the appearance, taste, aroma, and texture were the main quality parameters affecting consumer perception of the cooked rice [6,10,11]. A study conducted by Jeesan and Seo [12] showed that the color cues of cooked rice elicited consumer perceptions of the aroma, affected acceptance, and evoked a range of emotional responses.

Rice-quality assessment is an essential routine in rice production to maintain high-quality rice in the market and ensure high consumer acceptability. Standards for rice milling quality have been established to promote rice trading and marketing. According to the United States Standards for Milled Rice [13], rice is graded into categories considering (i) the maximum limits of the paddy kernel; (ii) the red rice; (iii) the chalky kernel; (iv) the broken kernel and other types of rice; (v) the color requirements of milled rice; and (vi) the minimum milling requirement. Besides, the Ministry of Agriculture in China established the standard for milling quality (NY/T593-2013) to improve rice grain quality production, emphasizing the standard requirements for different rice types such as *indica* and *japonica* rice [14].

Rice quality is commonly determined through visual inspections and manual measurements [1]. However, this approach is time-consuming, subjective, and prone to human error. Currently, there are analytical instruments based on imaging techniques to automate rice quality assessment, such as the Image-Rice Grain Scanner (Selgron, Blumenau, Brazil) and the SeedCount SC5000 Image Analyzer (Next Instruments, Condell Park, City of Canterbury-Bankstown, NSW, Australia). The Image-Rice Grain Scanner (Selgron, Blumenau, Brazil) provides 39 outputs of rice grain traits, including grain size, chalkiness, grain defect, and milling quality based on three-dimensional measurement of the rice grain images obtained from a camera [15]. Hence, it enables the rice breeder to select the desired rice quality traits in a shorter time with high accuracy. Furthermore, the SeedCount SC5000 Image Analyzer was developed using a flatbed scanning system in reflectance mode to obtain the individual rice sample images to measure the grain dimensions, chalkiness degree, and elongation [16]. For instance, it was used to determine the length, width, and length-to-width ratio of Australian wild rice [17] and chalkiness percentage in rice grains [18,19]. However, the lab-based instruments are costly and may hinder their application among small enterprises, especially in developing countries. Therefore, it is important to develop an alternative method using a rapid, reliable, cost-effective, and less complicated approach.

Emerging technologies such as computer vision (CV) and machine learning (ML) techniques have been applied to classify images of rice varieties [20], whole and broken rice grains [21], chalky rice [22,23], and discolored rice [24]. This technique requires the image acquisition of rice samples and computer vision algorithms to pre-process, analyze, and extract valuable information from the images to develop the classification models. Software, such as Matlab (Mathworks, Inc. Natick, MA, USA) [25,26,27] and LabVIEW (National Instruments, Austin, TX, USA) [28,29,30] and open-source libraries, such as OpenCV (Intel, Santa Clara, CA, USA) [31,32,33] and Python (Python Software Foundation, Wilmington, DE, USA) [34] are some of the most popular used among researchers. The artificial neural networks (ANNs) for supervised ML are well-known for solving multiclass classifications due to their ability to deal with non-linear data for pattern recognition to obtain high accuracy. For example, the ANN models were used in previous studies to classify mulberry fruit according to the ripeness levels [27], detect beer faults using the electronic nose [35], and classify aphid infestation levels using the electronic nose and near-infrared spectroscopy [36].

Rice from different cultivars differs in its physicochemical properties [4,37,38]. The morpho-colorimetric parameters, such as the grain’s major and minor axis length; aspect ratio; perimeter; eccentricity; roundness; red, green, and blue (RGB); and CIELab color spaces are key parameters that can be extracted using computer vision techniques. These have been used in previous studies, for example, to classify rice grains according to low-, medium-, and high-quality [39] and sound, broken, discolored, un-husked paddy, deformed, and withered grains [24]. Besides, the fractal dimension (FD) obtained by implementing the box-counting method has been used in previous research to classify grapevine leaves of different cultivars [40], characterize pork loin, and salmon sliced tissue [41], and analyze the microstructure of baked food products [42]. Hence, the FD could also be used as a critical input parameter to classify rice grains morphometrically. Nevertheless, the work on classifying rice using dimensionless parameters extracted automatically from digital images has not been much reported, especially for an extensive range of commercial rice samples. Moreover, little work has been conducted to evaluate the deployment accuracy of different conditions based on the developed model, resulting in a robust classification model.

Recent advancements in new-generation smartphones with high-resolution cameras, built-in sensors, powerful processors, and high-rate data transfer have enabled this technology adoption to be a cost-effective measurement and sensing tool. Hence, smartphone-based applications with computer vision algorithms for agriculture and food sciences have gained attention among researchers. These kinds of applications include the VitiCanopy (The University of Adelaide, Adelaide, Australia), which allows winegrowers to estimate the canopy vigor and porosity of the grapevines [43]; Plantix (PEAT GmbH, Berlin, Germany) used to diagnose pests, diseases, and nutritional deficiencies in 30 types of crops [44]; and FruitSize (Central Queensland University, Queensland, Australia), used to measure fruit size obtained from images captured from a smartphone camera [45]. Moreover, the smartphone has been used in previous studies to, for example, capture rice grain images for moisture content estimation for in-field application at harvest [46], detect milk adulteration [32], assess dietary information based on food and drink images [47], and estimate leaf area index (LAI) and plant height for canopy structures [48], which could be further developed into a smartphone app. Therefore, smartphone technology advancement has great potential to enable on-site measurement and rapid analysis at a lower cost, especially for the agricultural and food industries.

This study presents a smartphone-based imaging system as a tool to acquire images, semi-automated CV algorithms, and ML for rapid assessment techniques to classify commercial rice grains. This study used 15 commercial rice images to extract the morpho-colorimetric parameters using the customized CV algorithms written in Matlab^®^ R2021a. The ML model based on pattern recognition of artificial neural networks (ANNs) was developed to classify the commercial rice samples using morpho-colorimetric parameters as inputs. The proposed method and the classification model were then retrained to deploy the model in a different imaging condition to simulate real-time application. The proposed method would form the foundation for a smartphone-based app as a viable alternative to the conventional approach, a mobile, cost-effective, and user-friendly tool for the rapid assessment of the rice quality traits associated with consumer perception.

## 2. Materials and Methods

### 2.1. Rice Samples

In this study, 15 commercial rice grain types were obtained from local retailers in Australia (Table 1). The samples consisted of two main categories of rice on the market: the white rice produced from whole-grain rice by a milling process to remove the outer bran layer of the grain and whole-grain (unpolished) rice. For each sample, 2 g of whole kernels were selected from each packet in triplicates, corresponding to a different total number of rice grains per type.

### 2.2. Image Acquisition

**Figure 1:**
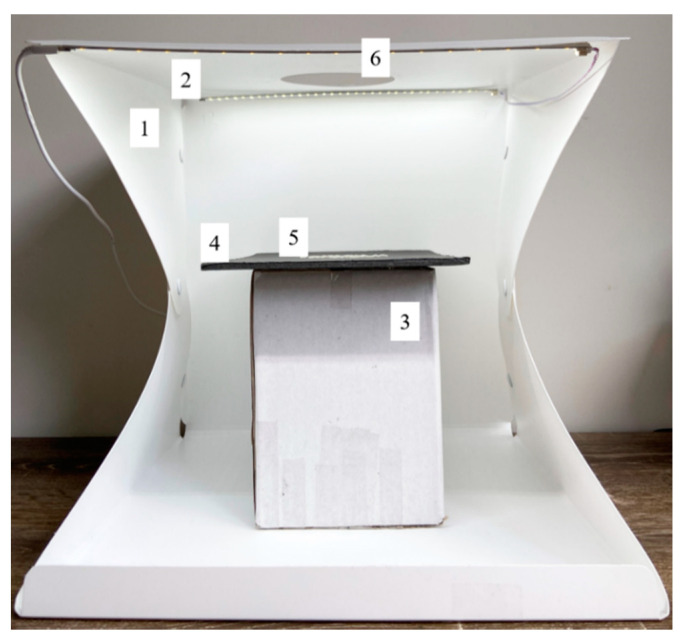
Experimental setup for image acquisition consisting of (**1**) a lightbox; (**2**) 70 pieces of two LED light strips; (**3**) a platform; (**4**) cardboard to place the grain for image acquisition; (**5**) rice samples; (**6**) top opening of the lightbox used to acquire the images using a smartphone. LED = light-emitting diode.

The images of rice samples were acquired using the rear camera (12-megapixel; focal length, *f* = 26 mm; aperture = *f*/1.8) of an iPhone 11 (Apple Inc., Cupertino, CA, USA). The Lightbox 1, a foldable lightbox tent with (Unbranded, Unhobest, China; dimensions: 40 cm × 40 cm × 40 cm), consisting of two daylight LED strips with 70 LEDs each, was used to acquire the images from the top opening at approximately 15 cm (Figure 1). The images were obtained via an Adobe Lightroom application (v6.1.0 Adobe Inc., San Jose, CA, USA) to allow a custom white balance using a white reference paper. A black background was used to capture the white and brown rice, and white background was used for the black and wild rice to provide good contrast between the foreground and background images. The rice samples were arranged in non-touching and random positions to minimize noise by touching and overlapping rice grains into the ANN modelling. However, the code includes watershed segmentation procedures for deployment to facilitate extraction of individual rice features even when touching each other or overlapping.

**Figure 2:**
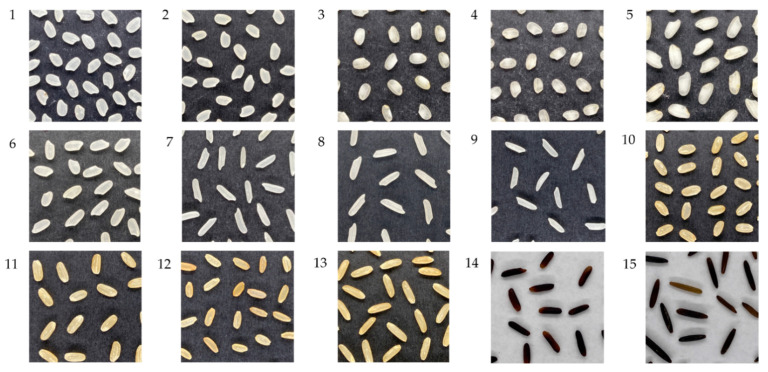
Examples of the 15 commercial rice sample images used in the study. The details of the rice samples based on the labeled class ID correspond to the list in Table 1. Rice sample images shown in the figure were cropped for presentation purposes only.

Figure 2 shows images obtained from each set of rice samples using the experimental setup. The images were acquired at a 3024 × 4032 pixels resolution and saved in Joint Photographic Group (JPG) format. Images captured using the smartphone were uploaded to the Matlab Drive^®^ through Matlab Mobile for further processing using Matlab^®^ R2021a on a personal computer (PC).

### 2.3. Image Pre-Processing, Segmentation and Extraction of Morpho-Colorimetric Features

**Figure 3:**
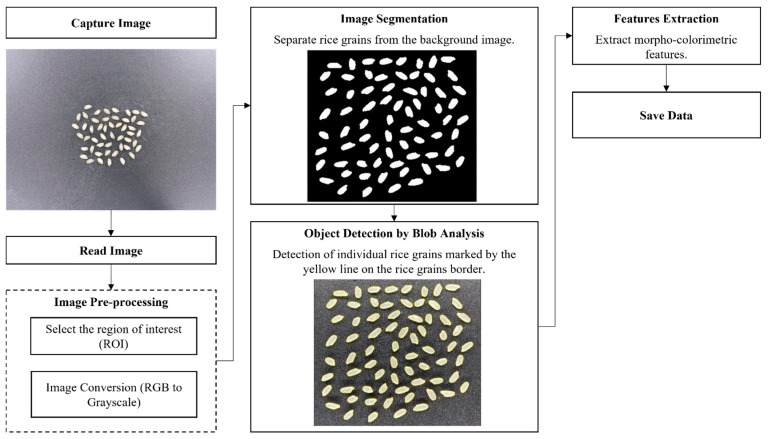
Flow diagram of the method used to extract the morpho-colorimetric features from the rice grain image.

The individual rice kernels were automatically analyzed to extract morpho-colorimetric features from the images using customized CV algorithms modified from previous work on leaf classification [25,40] in Matlab^®^ R2021a. The overview of the process is presented in Figure 3; it consists of the following steps: (i) image capturing and reading; (ii) image pre-processing; (iii) image analysis; and (iv) feature extraction to retrieve the morpho-colorimetric features of the rice grains.

### 2.4. Morpho-Colorimetric Parameters of Commercial Rice Grains

Table 2 shows the five morphometric and four colorimetric parameters extracted from the individual rice grain image. Unitless morphometric parameters were included in the study to simplify the image-capturing step and allow the user to independently capture the rice images in the lightbox at any object distance. The identification of single rice seeds was based on blob analysis from binarized images, which identifies contours of blobs to automatically label each grain to extract statistical shape measurements using the regionprops function in Matlab^®^ R2021a. From that analysis, automatic statistics are calculated, such as centroid, the aspect ratio and the area-parameter ratio index, computed using the major and minor axis length, area (A), and perimeter (P) of the rice images. The fractal dimension (FD) of the rice grains was determined using the box-counting method based on previously published work to recognize and analyze grapevine leaves [40] and medicinal plant leaves to extract similar features [25]. The binarized image of rice grains was also used as a mask on the original image to extract automatically colorimetric parameters from each rice grain of CIELab and RGB color scales. The yellowness index was computed from color scale parameters to determine the yellowness degree of the rice grains based on the previous work conducted by Rhim et al. [49]. The extracted features from the individual rice grains were automatically saved in Microsoft Excel Binary File Format (.xls) and were used as inputs for ML model development to classify the 15 commercial rice grains.

### 2.5. Statistical Analysis

A one-way analysis of variance (ANOVA; *p* < 0.05) and Tukey’s Honestly Significant Difference (HSD) post hoc test (α = 0.05) were conducted using Minitab 19.1 (Minitab Inc., State College, PA, USA). It was performed to assess whether there were significant differences between the means of the morpho-colorimetric parameters of the commercial rice samples obtained using Lightbox 1.

Multivariate data analysis based on the principal component analysis (PCA) and cluster analysis was conducted using a customized code written in Matlab^®^ R2021a. The main use of the PCA in this study was to find relationships between variables and samples as they are constructed using covariance methods as a parameter engineering justification for the ANN modelling presented [51,52,53,54,55]. Besides, cluster analysis helps to visualize the relative grouping of commercial rice samples according to these parameters. This type of analysis to support parameter engineering has been used in several ANN works for food and beverage applications [56,57,58] and helps non-experts in AI or machine-learning understand better the relationships between different parameters from the physicochemical point of view. This type of multivariate data analysis also helps to clarify the “black-box” properties of supervised machine learning such as ANN and to visualize that ANN correctly estimates the targets and that they are not artifacts from non-related inputs.

### 2.6. Machine Learning Modeling

The classification ML models were developed using the ANN algorithm for pattern recognition using a customized code written in Matlab^®^ R2021a. The model was developed by testing 17 training algorithms (data not shown), which consisted of three types of main functions: (i) backpropagation with Jacobian derivatives; (ii) backpropagation with gradient derivatives; and (iii) supervised weight and bias training functions [59]. The optimum classification algorithm was then selected by assessing the accuracy and performance of the model, indicated based on mean squared error (MSE); to assess any signs of under- or over-fitting, the MSE value for the training stage must be lower than the value for the testing stage. Furthermore, the number of inputs must be <70% of the number of samples to avoid over-fitting, which this model meets with only nine inputs [60,61,62]. Furthermore, the receiver operating characteristic (ROC) curves were used to analyze the model’s sensitivity (true positive rate) and specificity (true negative rate) to classify each type of rice [63]. A neuron trimming exercise was conducted using ten, seven, five, and three hidden neurons to find the optimal neuron number, followed by retraining the model several times to assess the consistency of the results and find the best model based on accuracy and performance. The number of neurons must also be considered to assess under- or over-fitting; a larger number of neurons, usually >10, is more likely to lead to over-fitting, while a very low number, usually below three, may lead to under-fitting [56].

**Figure 4:**
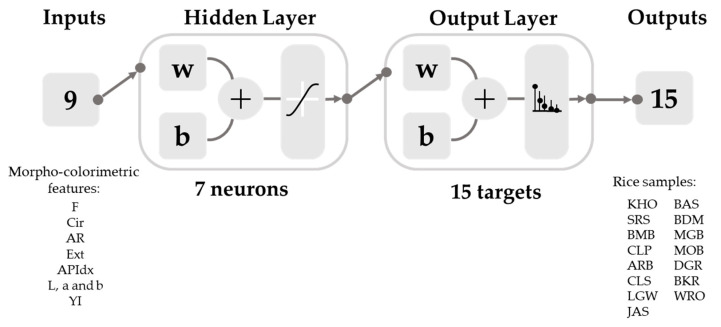
Diagram of a neural network model (Model 1) of the Bayesian Regularization algorithm with seven hidden neurons and sigmoid function showing nine inputs of morpho-colorimetric parameters and 15 outputs of commercial rice grains. The abbreviations for the morpho-colorimetric parameters (inputs) and commercial rice grains (outputs) are shown in Table 1 and Table 2. w = weight; b = bias.

In this study, Model 1 was developed using a data set extracted from the rice images obtained from Lightbox 1. After the screening, the Bayesian Regularization (BR) with seven neurons was selected because the model presented high accuracy, best performance, and no under- or over-fitting signs. The model thus consisted of a two-layer feedforward neural network with a sigmoid function using nine morpho-colorimetric parameters as inputs to classify the grains according to 15 types of commercial rice (Figure 4). The data set from the population of the rice grain samples for training and testing was randomly divided into 70% (*n* = 2687) and 30% (*n* = 1152), respectively.

### 2.7. Retraining and Deployment of the Machine Learning Model

A test was conducted to evaluate the deployment of the proposed method to simulate the practical application in a different condition. Therefore, a new data set corresponding to the 15 rice samples was acquired using Lightbox 2 (2D PhotoBench 120, Ortery Technologies Inc., Irvine, CA, USA; Dimension = 61.0 cm × 61.0 cm × 71.1 cm) with 5700 K daylight LED lighting, using similar approaches to those described in Section 2.2 with slight modification. The same smartphone was attached on a mini tripod at 15 cm from the samples. The images were acquired using Adobe Lightroom to set a custom white balance using white reference paper. Data from eight replicate images containing around 50 rice grains in an image were acquired and extracted from the customized CV codes described in Section 2.3.

**Figure 5:**
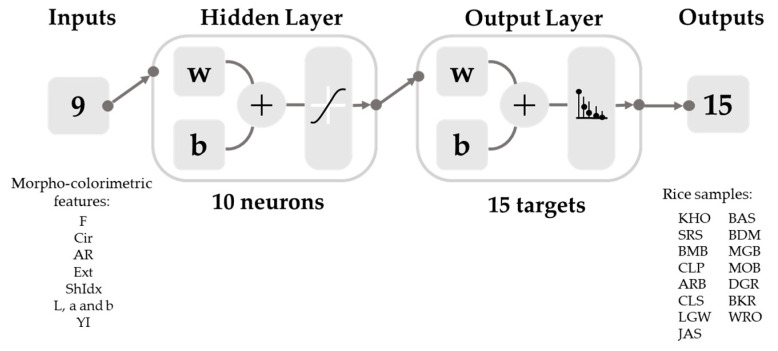
Diagram of a neural network model (Model 2) of the Bayesian Regularization algorithm with seven hidden neurons and sigmoid function showing nine inputs of morpho-colorimetric parameters and 15 outputs of commercial rice grains. The abbreviation for morpho-colorimetric parameters (inputs) and commercial rice grains (outputs) are shown in Table 1 and Table 2. w = weight; b = bias.

The new data from Lightbox 2 were fed to retrain the original Model 1 using the steps mentioned earlier in Section 2.6; the retrained Model 1 was then named Model 2. It was developed based on the nine morpho-colorimetric parameters as inputs to classify 15 commercial rice grains as targets, similar to the original Model 1 using a random data division of 70% (*n* = 6887) for training and 30% (*n* = 2952) for testing data sets (Figure 5). After testing the model on the 17 ANN algorithms, followed by the neuron trimming test, the best model was obtained using the BR algorithms with a sigmoid activation function and ten hidden neurons.

**Figure 6:**
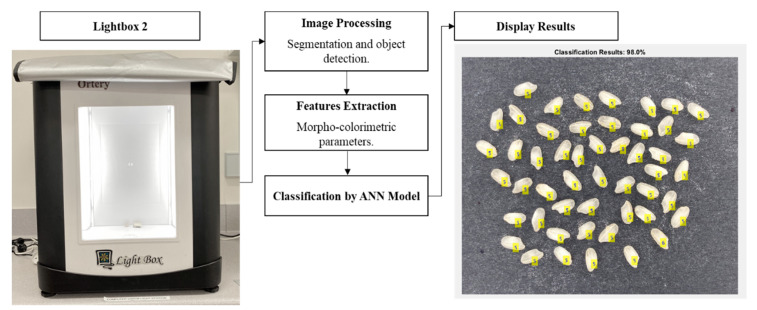
Flow diagram of the rice classification using computer vision and machine learning analysis. ANN = artificial neural network.

Deployment accuracy was tested using a new image captured for each type of rice obtained from Lightbox 2 using the same procedure as those used to develop Model 2. The image acquired using the smartphone was sent to Matlab Drive^®^, followed by the following steps to process the retrieved image in a laptop computer to detect individual rice grains, extract morpho-colorimetric features, and classify them according to their corresponding class ID using the developed ML model embedded in the code. Finally, a decision image was displayed with the labeled predicted class ID for each rice grain in the image. Figure 6 shows the flow diagram of the rice classification process.

## 3. Results

### 3.1. Morpho-Colorimetric Parameters of Commercial Rice Grains

Table 3 shows the ANOVA results of the morpho-colorimetric parameters from the dataset obtained using the foldable lightbox tent for each type of rice. Significant differences (*p* < 0.05) were observed between the samples in all parameters. The mean values for the FD obtained ranged from 1.57 (LGW) to 1.83 (BMB). The FD values were higher among the short-grain samples (the KHO, the SRS, the BMB, and the CLP), while medium-grain samples mainly had intermediate FD values.

The WRO had the lowest mean for Cir (0.46), while the short-grain rice samples, such as the CLP (0.91) and the BMB (0.91), were among the samples with high mean values. The AR is the ratio between the major and minor axis length and showed low mean values among the short-grain rice samples (1.63–1.97) compared to the medium- and long-grain rice samples (2.06–5.19). The short-grain rice samples such as the CLP (0.74), the BMB (0.73), the KHO (0.72), and the SRS (0.72) were the rice samples with high mean values for Ext, and the WRO (0.45) was the rice sample with the lowest Ext. The APIdx calculated using the pixel area, and the perimeter ratio showed that the BAS (0.27) had the lowest mean value among rice samples. Among all the samples, the WRO had a high AR value and low values for Cir, Ext, and APIdx, showing that its size and shape were different from the other types of rice, which are very long and narrow, reflecting its characteristics as a long and slender-shaped grain.

The L mean value was higher for the white rice samples, such as the JAS (59.46), the LGW (59.38), and the BAS (59.16), and lower L values were obtained for highly pigmented rice, such as the BKR and the WRO (27.54 and 29.60, respectively). As opposed to the L, the YI for the WRO and the BKR was higher than the white rice samples. The inverse trends for the L and YI parameters described the rice grain’s lightness and yellowness, respectively. A low positive value was observed in mean values of *a* for all white rice samples, and higher mean values were obtained for both highly pigmented rice samples. The unpolished rice samples had a higher *b* value compared to the white rice samples.

### 3.2. Multivariate Data Analysis

**Figure 7:**
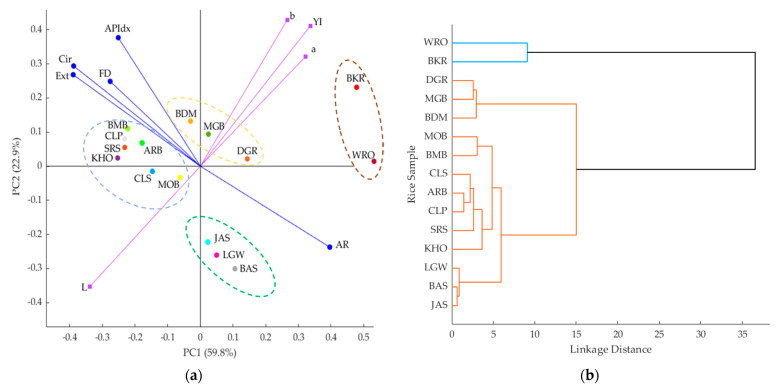
The multivariate data analysis for (**a**) principal component analysis (PCA) biplot for morpho-colorimetric parameters of 17 commercial rice types, where PC1 = principal component one, PC2 = principal component two; and (**b**) cluster analysis of the commercial rice samples based on morpho-colorimetric parameters. The abbreviations for morpho-colorimetric parameters and rice samples are shown in Table 1 and Table 2.

Figure 7a shows the PCA biplot for the nine morpho-colorimetric parameters and the 15 commercial rice samples from the data set obtained from the foldable light box tent. The PCA explained the 82.7% total data variability (PC1: 59.8%; PC2: 22.90%). Based on factor loadings (FL), principal component one (PC1) was characterized by AR (FL = 0.40) and YI (FL = 0.34) on the positive side of the axis, whereas Ext (FL = −0.39), Cir (FL = −0.39), and L (FL =−0.34) represented PC1 on the negative side. The principal component two (PC2) was mainly represented by *b* (FL = 0.43) and Y1 (FL = 0.41) on the positive side, and L (FL = −0.35) on the negative side.

The colorimetric parameters, such as YI, *b*, and *a,* were positively related and associated with the BKR and, to a lesser extent, the brown rice samples, such as the MGB, the DGR, and the BDM, which are located in the center of the PCA. In contrast, the L was negatively related to the latter parameters and associated with the MOB (brown rice). The morphometric parameters such as Ext, Cir, FD, and APIdx had a positive relationship and were associated with most rice samples belonging to the short and medium grains. Parameter AR was negatively related to the latter parameters and associated with the long-grain rice (JAS, LGW, and BAS).

Figure 7b shows the cluster analysis using the Euclidean linkage of PCA based on the nine morpho-colorimetric parameters to group the rice. The unpolished rice, the WRO, and the BKR were found in the same group, while the rest of the rice samples were clustered in a group. Likewise, Figure 7a showed groups of rice samples identified for black rice (BKR) and wild rice (WRO), brown rice (DGR, MGB, and BDM), and long-grain (JAS, LGW, and BAS) rice. However, there was no clear distinction between short-grain (BMB, CLP, KHO, and SRS) and medium-grain (ARB and CLS) rice. 

### 3.3. Machine Learning Modelling

Table 4 shows the statistical results of classifying the commercial rice grains based on nine morpho-colorimetric parameters using the Bayesian Regularization algorithm. Both classification models had high overall accuracies (>90%), with a lower MSE value for training (MSE < 0.01) than testing (MSE = 0.01) stages. Moreover, comparable accuracy was obtained for the training and testing stage for both models. This showed that the models had no signs of under- or over-fitting.

**Figure 8:**
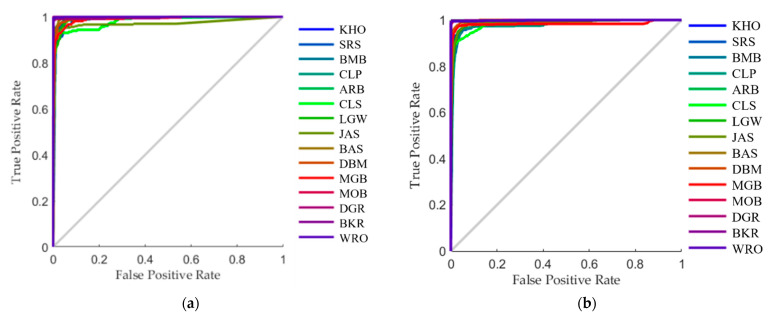
The receiver operating characteristic (ROC) curve of the artificial neural network models to classify 15 commercial rice samples using morpho-colorimetric parameters as inputs for (**a**) Model 1 and (**b**) Model 2. The abbreviations for rice samples are shown in Table 1.

Figure 8 shows the receiver operating characteristic (ROC) curve of the true-positive (sensitivity) versus the false-positive rates for both ANN models for classifying commercial rice grains. Based on the plot, the curves are closer to the true-positive rate at the y-axis, showing that the classification model had high true-positive rates (sensitivity) for classifying the rice samples correctly. It also showed that the models had a high predictive power to classify into each type of rice.

### 3.4. Evaluation of Commercial Rice Grains Classification Using ML

The deployment accuracies of the developed models for classifying new data sets of rice samples are shown in Table 5. The model’s performance in classifying the rice samples (*n* = 50) showed a high mean accuracy of 93.9%. The results also showed that the model successfully classified all the rice samples according to their respective rice class with ≥82%, which is acceptable for the application.

## 4. Discussion

### 4.1. Morpho-Colorimetric Features

The developed codes to extract nine morpho-colorimetric parameters were modified from previous works on leaf classifications for grapevine cultivars [40] and Chinese medicinal plants [25]. The novelty in this work was the incorporation of dimensionless morphological parameters, so the distance of the camera from the rice grains can vary with different setups. For fractal analysis parameters, the FD was formerly used by Jinorose et al. [64] to examine the effect of the parboiling process and cooking time on the physical changes of cooked rice grains based on image analysis. The morphometric parameters could discriminate short-grain rice groups indicated by high mean values for FD, Cir, Ext, APIdx, and low AR. Moreover, Cir and AR showed their potential as key parameters for ML modeling in classifying the ARB and the CLS under a similar grouping. This is in accordance with Calingacion et al. [37], who suggested that these types of rice were categorized as medium-length with bold-shape grains.

The colorimetric parameters extracted from the rice showed that white rice and unpolished rice vary because of the pigmentation on the bran layer of unpolished grain. Besides, the ARB, the CLP, and the BMB rice samples used for risotto and paella have chalky kernels that are more opaque than the translucent rice cultivars such as the LGW, JOM, and JSR [65]. High pigmentation on the BKR and the WRO rice samples discriminated by high *a* (redness) value were in accordance with previous research, which suggested that the pigmentation for those types of rice was related to the reddish color on the rice grains [17,66]. The high pigmentation corresponds to the carotenoid and anthocyanin content, which is linked with significant health benefits [67]. It is known that different colors of rice grains may depend on several factors, such as the varieties, the milling degree, the aging, and the parboiling process. A previous study showed that the relationship between the chalkiness and the physicochemical properties in rice was reflected by high total-starch accumulation, low total protein, and amino acids in the chalky part [68]. Besides, the milling process to polish the brown rice by removing the bran layer for white rice production could also affect the color variation in rice grains because different types of rice may be polished at different milling degrees during the process [21,69]. Therefore, the significant differences in grain pigmentation were considered relevant parameters to develop the classification model when using supervised ML in the parameter engineering process of supervised ML modeling.

### 4.2. Multivariate Data Analysis

Based on PCA and cluster analysis, it was found that most short- and medium-grain rice samples were grouped, as shown by the high similarities among the rice samples, which could be explained by their positive associations with FD, Cir, and Ext and negative associations with AR. Commonly, the AR is used to categorize the rice into three shape classes: bold (<2), medium (2.1–3), and slender (>3) [70]. This could be explained by previous research conducted by Calingacion et al. [37], in which both short- and medium-grain rice may have bold- and medium-shaped rice. As the PCA and cluster analysis showed an unclear separation among the rice samples, it was important to explore the potential of machine learning modeling to classify the rice.

### 4.3. Machine Learning Modeling

For ML models developed in this study, comparable MSE values were obtained for the training and testing stage, implying that the developed model showed no signs of over-fitting [71]. A similar finding from previous work was reported based on a comparative empirical study between BR and LM algorithms to develop the ANN model for social data prediction. The BR showed better performance than the LM algorithm for data prediction and supported its performance in dealing with high complexity data [62].

A previous study reported the classification of three types of commercial Basmati rice images using a k-Nearest Neighbor (k-NN) classifier based on morphometric parameters, such as the area, major axis, minor axis, eccentricity, and perimeter [72]. However, the overall classification accuracy was 79%, which could lead to poor estimation during deployment. Moreover, using specific dimensions from rice may jeopardize the accuracy of models using different settings, especially camera distance from the objects of interest. In contrast, Anami et al. [73] compared classification models developed using the Multilayer Back Propagation Neural Network (BPNN), the Support Vector Machine (SVM), and k-NN to classify five levels of adulteration in bulk paddy grain. The latter model was developed using the combination of color and texture parameters extracted from the images. The BPNN model was identified as the best model to classify the rice adulteration level at an average of 93.31%. However, it required a high number of input data (40 principal component coefficients) compared to the present study, which used only nine easily derived morpho-colorimetric inputs to obtain a comparable accuracy. Therefore, this study demonstrates the importance of selecting the appropriate inputs, classifiers, training algorithms, and hyperparameters to optimize the classification accuracy, which may help avoid under- and over-fitting and mitigate the high computational requirements.

The default data division of 70% for training and 30% for testing was used in the study as they represented a sufficient number of samples in each category. This method uses independent sets of samples for each stage and evaluates the overall accuracy by including all samples. A similar data division to develop ANN models was used in previous studies [35,59,74]. Besides, several retraining attempts were conducted to assess the consistency of the results, obtaining similar results in every attempt. Furthermore, the deployment using new data further validates the accuracy and performance of the model.

### 4.4. Deployment of the Classification Machine Learning Model

One of the challenges of supporting practical application in a different controlled environment is the high sensitivity to lighting conditions and camera settings, contributing to high misclassification when testing on a new data set obtained under different conditions. Therefore, Model 2 was developed using the new data sets obtained from Lightbox 2 to evaluate the deployment accuracy of the initial Model 1. The high deployment accuracy showed that the developed model based on adimensional morphological parameters is robust and reliable. Unlike the existing classification models, the ANN model developed in this study included a comprehensive and complex range of commercial rice samples as targets, including white, brown, black, and wild rice. Therefore, the model appears to exhibit robustness in classifying the different commercial rice grains available globally in the market.

The advantage of using dimensionless parameters is that the user is allowed to capture images without strict settings. The demonstrated method is independent of the type of camera and the camera’s distance from the object. Moreover, the classification model was developed using two lightbox systems, increasing model generalizability and adaptability to new data.

The work in this study is highly significant to the rice industry as the extracted morpho-colorimetric parameters were associated with consumer perceptions of raw [6,7,8,9] and cooked rice [6,10,11] quality. Therefore, these parameters could predict consumer perceptions of rice associated with the appearance quality traits for rice types. Furthermore, the automatic extraction of features and ANN modelling will help the industry to certify rice types and prevent adulteration [75]; furthermore, the ANN modelling based on feature extraction as inputs could also be used to target consumer perception and quality parameters as it has been performed for other food and beverage products [57,59,76].

This grain-by-grain scale approach proposed for quality assurance may avoid manual analysis and destructive assessment and save time compared to traditional descriptive sensory analysis with trained panelists. The effort of utilizing a smartphone camera to capture the images paired with a semi-automated CV algorithm could accelerate this development, making it cost-effective, user-friendly, rapid, and convenient. Moreover, since the smartphone is portable, it can also support on-site assessment instead of using specialized equipment in the laboratory.

## 5. Conclusions

This study showed the development of a cost-effective and rapid method to classify commercial rice samples obtained from a smartphone camera. It was achieved by integrating CV algorithms to extract morpho-colorimetric parameters and ML to classify 15 types of commercial rice grains. High classification accuracies were obtained based on ML models developed using the dimensionless parameters as inputs captured from different lightboxes, which increases model generalization. Further studies are required to link these easily obtained parameters with other quality traits and compositional parameters of rice grains that are important for the industry. Moreover, the methodologies proposed in this study can be applied by the industry to develop a smartphone application integrated with cloud-based computing to automatically assess consumer perception associated with rice quality traits and in real-time. The latter is achieved by acquiring consumer sensory perceptions through images of rice and cooked rice. This can benefit the industry in monitoring rice quality conveniently along the rice supply chains as well as at the consumer end.

## Figures and Tables

**Table 1:** Details of commercial rice samples, including class ID, product category, type, origin, abbreviation, and the number of grains per rice type in triplicates obtained using Lightbox 1.

Class ID	Product Category	Type	Origin	Abbreviation	Number of Grains
1	White rice (Polished rice)	Khoshihikari ^a^	Japan	KHO	243
2		Sushi rice ^a^	Japan	SRS	276
3		Bomba ^a^	Spain	BMB	210
4		Calasparra ^a^	Spain	CLP	263
5		Arborio ^b^	Italy	ARB	126
6		Calrose ^b^	Australia	CLS	230
7		Long-grain ^c^	Thailand	LGW	195
8		Jasmine ^c^	Thailand	JAS	297
9		Basmati ^c^	Pakistan	BAS	330
10	Whole grain rice (Unpolished rice)	Biodynamic rice ^b^	Australia	BDM	224
11		Medium grain ^b^	Australia	MGB	212
12		Medium-grain—organic ^b^	India	MOB	274
13		Doongara ^c^	Australia	DGR	308
14		Black rice ^c^	Thailand	BKR	317
15		Wild rice—organic ^c^	USA	WRO	335

^a^ short-grain; ^b^ medium-grain; ^c^ long-grain.

**Table 2:** The adimensional morpho-colorimetric features and indices that were used to develop the ML (machine learning) model to classify 15 types of commercial rice grains.

Parameters	Abbreviation	Description
Fractal dimension	FD	Fractal dimension obtained from the box-counting method [40].
Circularity	Cir	Degree of object roundness, which returned the value between 0 to 1. The value 1 indicates a perfect circle.
Aspect ratio	AR	The ratio between major and minor axis length [50].
Extent	Ext	The ratio between the rice grain area and bounding box area.
Area-Perimeter Ratio Index	APIdx	APIdx = [(A/P) − (A/P)min]/(A/P)max
CIELab color scale	L, a and b	Lightness (L), red to green color range (a), and yellow to blue color range (b) [40].
Yellowness Index	YI	Degree of yellowness [49].

**Table 3:** The mean and ± standard error (SE) values for morpho-colorimetric parameters of the commercial rice samples.

Rice Sample	n	FD	SE	Cir	SE	AR	SE	Ext	SE	APIdx	SE	L	SE	*a*	SE	*b*	SE	YI	SE
KHO	243	1.82 ^a^	±0.08	0.89 ^b^	±0.02	1.63 ^h^	±0.09	0.72 ^a^	±0.04	0.70 ^c,d^	±0.08	55.96 ^d^	±1.61	1.93 ^e^	±0.55	−1.61 ^f^	±1.81	−4.17 ^i^	±4.69
SRS	276	1.82 ^a^	±0.08	0.90 ^a,b^	±0.02	1.64 ^h^	±0.10	0.72 ^a,b^	±0.04	0.71 ^c,d^	±0.08	53.80 ^f^	±1.81	2.05 ^e^	±0.17	−0.91 ^e^	±0.42	−2.42 ^g,h^	±1.12
BMB	210	1.83 ^a^	±0.10	0.91 ^a,b^	±0.04	1.61 ^h^	±0.11	0.73 ^a^	±0.05	0.75 ^c^	±0.20	55.20 ^e^	±1.75	1.89 ^e^	±0.38	0.70 ^c^	±0.65	1.78 ^e,f^	±1.64
CLP	262	1.82 ^a^	±0.12	0.91 ^a^	±0.08	1.58 ^h^	±0.16	0.74 ^a^	±0.05	0.69 ^d^	±0.31	54.62 ^e^	±2.09	2.52 ^c^	±0.37	−0.34 ^d^	±0.40	−0.88 ^g^	±1.05
ARB	126	1.66 ^c,d^	±0.04	0.83 ^c^	±0.02	1.94 ^g^	±0.10	0.67 ^c^	±0.05	1.17 ^a^	±0.10	54.87 ^e^	±1.71	1.34 ^f,g^	±0.31	−0.16 ^d^	±0.82	−0.40 ^f,g^	±2.16
CLS	230	1.67 ^c^	±0.18	0.83 ^c^	±0.04	1.97 ^g^	±0.20	0.68 ^c^	±0.07	0.75 ^c^	±0.07	56.71 ^c^	±3.31	1.27 ^g^	±0.52	−0.35 ^d^	±1.01	−0.91 ^g^	±2.52
LGW	195	1.57 ^f^	±0.10	0.61 ^g^	±0.04	3.32 ^c^	±0.34	0.57 ^d,e^	±0.13	0.46 ^f^	±0.08	59.39 ^a^	±1.06	1.31 ^g^	±0.19	−1.82 ^f^	±0.31	−4.38 ^i^	±0.76
JAS	297	1.59 ^e,f^	±0.10	0.64 ^f^	±0.09	3.18 ^d^	±0.47	0.59 ^d^	±0.14	0.50 ^f^	±0.21	59.50 ^a^	±3.41	1.30 ^g^	±0.40	−1.43 ^f^	±0.36	−3.56 ^h,i^	±0.50
BAS	330	1.58 ^f^	±0.17	0.59 ^h^	±0.11	3.59 ^b^	±0.66	0.55 ^e^	±0.15	0.27 ^h^	±0.18	59.16 ^a^	±1.47	1.52 ^f^	±0.25	−1.53 ^f^	±1.60	−3.71 ^h,i^	±3.87
BDM	224	1.62 ^d,e^	±0.16	0.82 ^c^	±0.03	2.06 ^g^	±0.18	0.69 ^b,c^	±0.08	0.83 ^b^	±0.11	53.10 ^g^	±1.93	2.27 ^d^	±0.30	3.91 ^b^	±1.03	10.55 ^d^	±2.88
MGB	212	1.59 ^e,f^	±0.12	0.79 ^d^	±0.06	2.27 ^f^	±0.26	0.6 ^c^	±0.10	0.71 ^c,d^	±0.29	55.20 ^e^	±1.46	1.97 ^e^	±0.27	4.72 ^a^	±2.02	12.22 ^c,d^	±5.23
MOB	274	1.71 ^b^	±0.17	0.78 ^d^	±0.06	2.33 ^f^	±0.31	0.67 ^c^	±0.10	0.49 ^f^	±0.11	58.01 ^b^	±0.97	1.99 ^e^	±0.20	1.08 ^c^	±0.63	2.67 ^e^	±1.57
DGR	308	1.59 ^e,f^	±0.09	0.67 ^e^	±0.03	3.06 ^e^	±0.23	0.60 ^d^	±0.12	0.63 ^e^	±0.09	52.99 ^g^	±1.65	1.51 ^f^	±0.29	4.86 ^a^	±1.81	13.18 ^c^	±5.09
BKR	317	1.59 ^e,f^	±0.09	0.60 ^g,h^	±0.05	3.56 ^b^	±0.39	0.56 ^e^	±0.14	0.50 ^f^	±0.11	27.54 ^i^	±2.22	14.64 ^a^	±1.32	4.97 ^a^	±2.35	25.11 ^a^	±10.43
WRO	335	1.66 ^c^	±0.12	0.46 ^i^	±0.05	5.19 ^a^	±0.82	0.45 ^f^	±0.16	0.39 ^g^	±0.12	29.60 ^h^	±2.32	7.95 ^b^	±0.94	4.19 ^b^	±2.88	19.59 ^b^	±13.50

Mean values with different letters for each parameter indicate significant differences based on ANOVA (*p* < 0.001) and Tukey’s honestly significant difference (HSD) test (α < 0.05). Abbreviations of samples are found in Table 1 and morpho-colorimetric parameters in Table 2.

**Table 4:** Statistical results of the ANN model using Bayesian Regularization algorithm of the artificial neural network.

Stage	Sample (n)	Accuracy (%)	Error (%)	Performance (MSE)
**Model 1 (Neurons = 7)**				
Training	2687	95.0	5.0	<0.01
Validation	-	-	-	-
Testing	1152	87.8	12.2	0.01
Overall	3839	92.9	7.1	-
**Model 2 (Neurons = 10)**				
Training	6887	91.6	8.4	<0.01
Validation	-	-	-	-
Testing	2952	88.5	11.5	0.01
Overall	9839	90.7	9.3	

**Table 5:** Results of deployment accuracy when tested on the new data set obtained from 2D PhotoBench 120 lightbox. The abbreviations for the rice samples are shown in Table 1.

Rice Sample	Class	Deployment Accuracy (%)
KHO	1	96.0
SRS	2	94.2
BMB	3	96.0
CLP	4	82.0
ARB	5	94.0
CLS	6	94.0
LGW	7	87.8
JAS	8	98.0
BAS	9	88.0
BDM	10	94.0
MGB	11	98.0
MOB	12	92.0
DGR	13	96.0
BKR	14	98.0
WRO	15	100.0
	Mean (%)	93.9

## Data Availability

Data and intellectual property belong to The University of Melbourne; any sharing needs to be evaluated and approved by the University.
